# Assessment of Social Behavior Using a Passive Monitoring App in Cognitively Normal and Cognitively Impaired Older Adults: Observational Study

**DOI:** 10.2196/33856

**Published:** 2022-05-20

**Authors:** Marijn Muurling, Lianne M Reus, Casper de Boer, Sterre C Wessels, Raj R Jagesar, Jacob A S Vorstman, Martien J H Kas, Pieter Jelle Visser

**Affiliations:** 1 Alzheimer Center Department of Neurology, Amsterdam Neuroscience Vrije Universiteit Amsterdam, Amsterdam University Medical Center Amsterdam Netherlands; 2 Department of Psychology Leiden University Leiden Netherlands; 3 Groningen Institute for Evolutionary Life Sciences University of Groningen Groningen Netherlands; 4 Department of Psychiatry The Hospital for Sick Children and University of Toronto Toronto, ON Canada; 5 Department of Psychiatry and Neuropsychology School for Mental Health and Neuroscience Maastricht University Maastricht Netherlands

**Keywords:** passive monitoring, smartphone app, cognitive impairment, social behavior, dementia, mHealth, mobile app, cognitive, mental health, social withdrawal, well-being

## Abstract

**Background:**

In people with cognitive impairment, loss of social interactions has a major impact on well-being. Therefore, patients would benefit from early detection of symptoms of social withdrawal. Current measurement techniques such as questionnaires are subjective and rely on recall, in contradiction to smartphone apps, which measure social behavior passively and objectively.

**Objective:**

This study uses the remote monitoring smartphone app Behapp to assess social behavior, and aims to investigate (1) the association between social behavior, demographic characteristics, and neuropsychiatric symptoms in cognitively normal (CN) older adults, and (2) if social behavior is altered in cognitively impaired (CI) participants. In addition, we explored in a subset of individuals the association between Behapp outcomes and neuropsychiatric symptoms.

**Methods:**

CN, subjective cognitive decline (SCD), and CI older adults installed the Behapp app on their own Android smartphone for 7 to 42 days. CI participants had a clinical diagnosis of mild cognitive impairment (MCI) or Alzheimer-type dementia. The app continuously measured communication events, app use and location. Neuropsychiatric Inventory (NPI) total scores were available for 20 SCD and 22 CI participants. Linear models were used to assess group differences on Behapp outcomes and to assess the association of Behapp outcomes with the NPI.

**Results:**

We included CN (n=209), SCD (n=55) and CI (n=22) participants. Older cognitively normal participants called less frequently and made less use of apps (*P*<.05). No sex effects were found. Compared to the CN and SCD groups, CI individuals called less unique contacts (*β*=–0.7 [SE 0.29], *P*=.049) and contacted the same contacts relatively more often (*β*=0.8 [SE 0.25], *P*=.004). They also made less use of apps (*β*=–0.83 [SE 0.25], *P*=.004). Higher total NPI scores were associated with further traveling (*β*=0.042 [SE 0.015], *P*=.03).

**Conclusions:**

CI individuals show reduced social activity, especially those activities that are related to repeated and unique behavior, as measured by the smartphone app Behapp. Neuropsychiatric symptoms seemed only marginally associated with social behavior as measured with Behapp. This research shows that the Behapp app is able to objectively and passively measure altered social behavior in a cognitively impaired population.

## Introduction

Alzheimer disease is a neurodegenerative disease that is pathologically characterized by abnormal amyloid and tau deposition [1]. The disease starts with a preclinical phase without any symptoms, and cognition and functional abilities decline over time toward the symptomatic stages of prodromal Alzheimer and Alzheimer-type dementia [1]. Social withdrawal, characterized by reduced social interaction and subjective feelings of loneliness [2], has been identified as one of the earliest symptoms of Alzheimer disease [3]. Alzheimer disease patients would benefit from early detection of symptoms of social withdrawal, since loss of social interactions is associated with accelerated symptom progression [3] and an increased risk of conversion to dementia [3-6].

Common methodology for assessment of social behavior is the use of clinical questionnaires such as the World Health Organization Disability Assessment Schedule [7] or Social Functioning Scale [8]. However, reliability of self-report questionnaires may be influenced by diminished social awareness in Alzheimer disease patients, depending on disease severity [9,10], while caregiver-reported questionnaires rely on recall and are burdensome and subjective. Consequently, self- or caregiver-reported clinical questionnaires on social behavior may not be a reliable tool for this particular patient group. Therefore, to detect symptoms of social withdrawal in Alzheimer disease, objective measures of social withdrawal are needed.

Smartphone apps are a potential tool for objective and passive assessment of social withdrawal. Advantages of smartphone apps include the possibility to collect large amounts of data in the natural environment of a participant, without the need for active involvement. The smartphone app Behapp [11] is designed to assess various aspects of behavior and includes measures such as call history, app use, and location that could be used as a proxy for social behavior [12]. In this study, we will therefore use the smartphone app Behapp to passively assess social behavior. Little information on social activities in older adults, both cognitively normal (CN) and cognitively impaired (CI), is available, and we will therefore also test the effect of factors that are known to influence social behavior, such as age, sex, education [13-15], and neuropsychiatric symptoms. Since Alzheimer disease patients often suffer from neuropsychiatric symptoms such as depression and apathy [16] and these symptoms might increase the risk of progressing to Alzheimer-type dementia [17-19], neuropsychiatric symptoms could consequently lead to increased social withdrawal.

The first aim of this research is to investigate the association between demographic characteristics and Behapp outcome variables in a CN control group. Second, this study aims to test if social behavior as measured using the passive smartphone app Behapp is altered in CI patients compared to 2 groups: CN older adults and people with subjective cognitive decline (SCD) [20], who are at risk of developing cognitive impairment [21]. Third, we will explore the association between the Behapp outcomes and neuropsychiatric symptoms as measured through the Neuropsychiatric Inventory (NPI).

## Methods

### Participants

We included 288 participants from 3 cohorts (Table 1): Hersenonderzoek.nl [22], the Amsterdam Dementia Cohort [23], and the Psychiatric Ratings Using Intermediate Stratified Markers (PRISM) study [24] (Multimedia Appendix 1, Table S1). For all participants, a minimum age of 45 years and minimum participation duration of 7 days were required. All participants owned an Android phone except for one participant, who received an Android phone for the duration of the study. Participants were included from 2017 to the beginning of 2020, before the start of the COVID-19 pandemic. Participants were assigned to group CN, SCD, or CI. All participants provided informed consent before participation in the study.

**Table 1:** Demographic characteristics of the 3 diagnostic groups.

		Total (n=288)	CN^a^ (n=209)	SCD^b^ (n=55)	CI^c^ (n=24)	Group comparisons^d^ (*P* value, difference)
Age (years), mean (SD)		63 (8)	63(8)	61 (7)	68 (8)	.002, CI>CN, CI>SCD
Female, n (%)		164 (56.9)	122 (58.4)	34 (61.8)	8 (33.3)	.046, CI<CN, CI<SCD
Education (years), mean (SD)		11 (3)	11 (2)	10 (2)	13 (5)	.003, CI>CN, CI>SCD
**Cohort, n (%)**						
	Hersenonderzoek.nl	232 (80.6)	195 (93.3)	36 (65.5)	1 (4.2)	—^e^
	ADC^f^	21 (7.3)	0 (0)	18 (32.7)	3 (12.5)	—
	PRISM^g^	28 (9.7)	14 (6.7)	0 (0)	14 (58.3)	—
	ADC + PRISM	7 (2.4)	0 (0)	1 (1.8)	6 (25.0)	—
App running time (days), mean (SD)		38 (9)	38 (9)	38 (9)	36 (11)	.78
NPI^h^ available, n (%)		41 (14)	0 (0)	19 (35)	22 (92)	—
NPI total score, mean (SD)		6.3 (8.5)	—	7.7 (11)	5 (5.6)	.97

^a^CN: cognitively normal.

^b^SCD: subjective cognitive decline.

^c^CI: cognitively impaired.

^d^Significant differences between the groups are shown in the last column: *P* values are given, and if *P*<.05, the group differences are given (eg, CI>CN meaning CI had higher mean than CN group).

^e^NA: not available.

^f^ADC: Amsterdam Dementia Cohort.

^g^PRISM: Psychiatric Ratings Using Intermediate Stratified Markers.

^h^NPI: Neuropsychiatric Inventory.

### Ethical Approval

Ethical approval was obtained before start of the study in both the Netherlands and Spain. All research centers in the Netherlands obtained ethical approval from the Ethical Review Board University Medical Centre of Utrecht (17-021/D) for the PRISM cohorts and from the Ethical Review Board VU University Medical Centre (2017.254) for the hersenonderzoek.nl and Amsterdam Dementia Cohort cohorts. In Spain, the PRISM study was approved by Comité Ético de Investigación Clínica Hospital General Universitario Gregorio Marañón (59359).

### Behapp App

Behapp is a smartphone app for Android phones, developed to objectively and passively measure sociability and social exploration [2,11]. Upon installation on the personal smartphone, each participant received an unique code to activate the app. Data collection via the app was set to stop automatically after 42 days.

After installation, Behapp continuously monitored measures of communication events (eg, incoming and outgoing phone calls), app activity (eg, social media or entertainment apps), and location via GPS. Data were encrypted before saving on the participants’ device and deleted immediately after uploading to the secured data server. Content of calls, SMS messages, and apps were not registered, collected, or saved by Behapp [25].

### Behapp Outcome Definitions

All Behapp outcomes are demonstrated in Table 2. For the calls category, the following definitions are used: unique contacts are the number of unique phone numbers from incoming or outgoing calls. Single use contacts are number of contacts called exactly once during the duration of the study. Mean repeated contacts are total number of calls divided by the number of unique contacts. The number of calls and duration of calls variables were divided by the number of days a participant participated in the research.

For the app use category, the following definitions are used: an app is open if it is running in the foreground. An app is opened if a participant brings the app to the foreground. Mean duration of opened apps is calculated as the total duration of the apps in the foreground during the duration of the study divided by the total number of times apps are opened during the duration of the study. Similar to the calls category, the number of times app opened variables were divided by the number of days a person participated in the research.

**Table 2:** Descriptive characteristics of each Behapp outcome for the cognitively normal group.

Category, subcategory, and variable				Median (25%-75%)		Age		Edu^a^		Missing data, n (%)^b^
**Calls**										
	**Incoming^c^**									
		Number	0.3 (0.1-0.7)		–^d^				3 (1)	
		Number of nonzero duration calls	0.6 (0.2-1.1)		–				3 (1)	
		Duration (s)	81.3 (17.7-177.4)		–				3 (1)	
		Number of unique contacts	0.2 (0.1-0.3)		–				3 (1)	
		Number of single use contacts	0.1 (0.1-0.2)		–				3 (1)	
	**Outgoing^c^**									
		Number	0.7 (0.3-1.4)						3 (1)	
		Duration (s)	79.5 (27.6-207.5)		–				3 (1)	
		Number of nonresponse calls	0.1 (0-0.3)						3 (1)	
		Number of unique contacts	0.3 (0.2-0.6)						3 (1)	
		Number of single use contacts	0.2 (0.1-0.3)						3 (1)	
	**Missed^c^**									
		Number	0.2 (0.1-0.3)		–				3 (1)	
		Number of unique contacts	0.1 (0-0.2)		–				3 (1)	
	**All**									
		Mean repeated contacts	2.6 (2-3.5)						3 (1)	
**App use**										
	**All^c^**									
		Number of times opened	86.1 (44.1-151.5)		–				10 (5)	
		Duration opened (s)	3743.1 (1821.6-7482)						10 (5)	
		Number of times opened at night	1.4 (0.2-4.8)						10 (5)	
	**Communication**									
		Number of times opened^c^	13.7 (6.5-26.3)		–		+^e^		10 (5)	
		Mean duration opened (s)	67.8 (50.7-86.4)		+				11 (5)	
	**Social media**									
		Number of times opened^c^	1 (0-4.4)		–				10 (5)	
		Mean duration opened (s)	104 (50.6-143.5)						71 (34)	
	**Entertainment**									
		Number of times opened^c^	0 (0-0.1)		–				10 (5)	
		Mean duration opened (s)	69.2 (27-138.4)						136 (65)	
	**News magazines**									
		Number of times opened^c^	0.5 (0-3.7)						10 (5)	
		Mean duration opened (s)	62.2 (31.9-118.9)						67 (32)	
**Location**										
	**Stay points**									
		Total number of stay points^c^	1.5 (1.1-2.3)						40 (19)	
		Total number of unique stay points^c^	0.4 (0.3-0.6)						40 (19)	
		Total number of nightly stay points excluding home^c^	0.1 (0-0.3)						40 (19)	
		Total number of unique nightly stay points^c^	0.1 (0-0.1)						40 (19)	
		Total number of outside office hours stay points^c^	0.3 (0.2-0.4)						40 (19)	
		Total number of unique outside office hours stay points^c^	0.2 (0.1-0.3)						40 (19)	
		Total number of single visits^c^	0.3 (0.2-0.4)				+		40 (19)	
		Percentage of stay points visited once	70 (60-77.8)						40 (19)	
		Mean time spent stationary (min)	838.8 (550.8-1208.2)						40 (19)	
	**Travel**									
		Mean distance traveled (km)	27.5 (17.7-44.3)						40 (19)	
		Standard deviation distance traveled (km)	34.9 (16-57)						40 (19)	
		Mean time traveled (min)	68.7 (51.7-96.8)						40 (19)	
		Standard deviation time traveled (min)	54.7 (40.8-90.6)						40 (19)	
		Total number of trajectories^c^	0.6 (0.1-1.2)						40 (19)	
		Maximum distance from home (km)	124.8 (64.3-301.6)						42 (20)	
		Average distance from home (km)	37.3 (19.4-90.9)						42 (20)	
	**Home**									
		Percentage of time spent at home	77.9 (64.4-88)						40 (19)	

^a^Edu: education.

^b^N and percentage of participants of whom the data for that specific variable is missing.

^c^Variables with values per day (total value divided by the number of days of participation).

^d^Indicates a significant negative association.

^e^Indicates a significant positive association.

For the location category, the following definitions are used: a stay point is a location based on GPS where a participant stayed for at least 60 minutes within a circle with radius 350 meters and center defined by the first measured location. Nightly stay points are stay points between midnight and 6 AM. Home is defined as the stay point where most time is spent between midnight and 6 AM during the duration of the study. Outside office hours stay points are any stay points except home, measured after 7 PM on weekdays and all day during the weekend. Mean time spent stationary is defined as the mean duration spent at stay points calculated from all stay points during the duration of the study. Again, the total number of stay points or trajectories variables were divided by the number of days a person participated in the research.

### CN Control Group

Participants in the CN group (n=209) did not report any memory complaints. They either self-registered online that they did not have any neurological or psychiatric diseases (n=195) or visited a memory clinic and scored approximately average on the Mini-Mental State Examination (MMSE) according to their age and years of education as compared with normative data (n=14). To find normal social behavior in cognitively normal older adults and to address our first aim to find possible associations between demographic characteristics and Behapp outcome variables in a cognitively healthy control group, this group was larger than the SCD and CI groups.

### Diagnostic Groups

Participants in the SCD group (n=55) self-reported memory complaints. The majority of this group (n=36) self-registered online and therefore were not neuropsychologically tested. The rest of this group (n=19) visited a memory clinic because of memory complaints but did not show objective cognitive deficits during neuropsychological testing [23].

Participants in the CI group (n=24) had a clinical syndrome diagnosis of either mild cognitive impairment (MCI; n=5) or Alzheimer disease dementia (n=19) [1]. Amyloid status was available from 5 participants, from which 4 participants were amyloid positive and 1 MCI participant was amyloid negative.

The outcomes of the Behapp app from the SCD and CI groups were compared with the CN group to address our second aim.

### Neuropsychiatric Symptoms

The NPI [26] is a caregiver-based instrument that measures the severity and frequency of neuropsychiatric symptoms, including delusions, hallucinations, agitation, depression, anxiety, euphoria, apathy, disinhibition, irritability, aberrant motor behavior, sleep dysfunction, and appetitive disturbances. The NPI was administered before the installation of the Behapp app. Outcomes were available for 20 SCD and 22 Alzheimer disease participants in the Amsterdam Dementia Cohort and PRISM cohort. Scores for each neuropsychiatric domain were derived by multiplying the severity score and frequency score from each domain. The total NPI score is the sum of all domain scores ranging from 0 to 144, with a higher score indicating more neuropsychiatric symptoms.

### Statistical Analyses

Statistical analyses were performed using R (version 4.0.2, R Foundation for Statistical Computing). Mann-Whitney *U* tests and Spearman rho were used to assess the association between the Behapp outcomes and demographic characteristics (ie, age, sex, and years of education) in the CN control group. Normality was tested using the Saphiro-Wilk test. Since the Behapp data were skewed, medians and quartile values are used to describe the data. Baseline characteristics of the CN, SCD, and CI groups were compared using analysis of variance, *t* test, Kruskal-Wallis test, or chi-square test, when appropriate.

Each Behapp outcome was logarithmically transformed to meet the normality assumptions and standardized to the control group by subtracting the mean of the control group and dividing by the standard deviation of the control group from each corresponding variable. There were no outliers that needed to be removed. Linear models were used with the standardized Behapp outcomes as dependent variable and group as independent variable, corrected for age, sex, and years of education. Regression models were used to examine associations between standardized Behapp outcome measures and the total NPI score, corrected for age, sex, and years of education. A *P*<.05 was considered significant. Assuming 3 clusters of Behapp outcomes (calls, app use, and location) in which the variables are highly correlated (Multimedia Appendix 1, Figure S1), all *P* values were corrected for 3 multiple comparisons using Bonferroni correction (*P* value/3). Since we were mainly interested in association patterns rather than individual relations, we decided not to reduce the number of variables.

## Results

### CN Control Group

The control group that did not experience any memory complaints consisted of 122 women and 87 men with a mean age of 62.7 years and a mean education of 10.6 years (Table 1). Descriptive characteristics for all Behapp outcomes can be found in Table 2. Older participants called less frequently and opened apps less frequently (Table 2). Individuals with a higher education opened communication apps more often and had a higher total number of single visits (Table 2). No differences were found between females and males.

### Diagnostic Groups

In total, 209 CN, 55 SCD, and 24 CI participants were included with an age range of 46 to 83 years. Demographic characteristics of the 3 groups can be found in Table 1. CI participants had the highest age (*P*=.002), highest years of education (*P*=.003), and fewest females (*P*=.046) compared to the CN and SCD groups. The number of measuring days did not differ between the groups.

**Figure 1:**
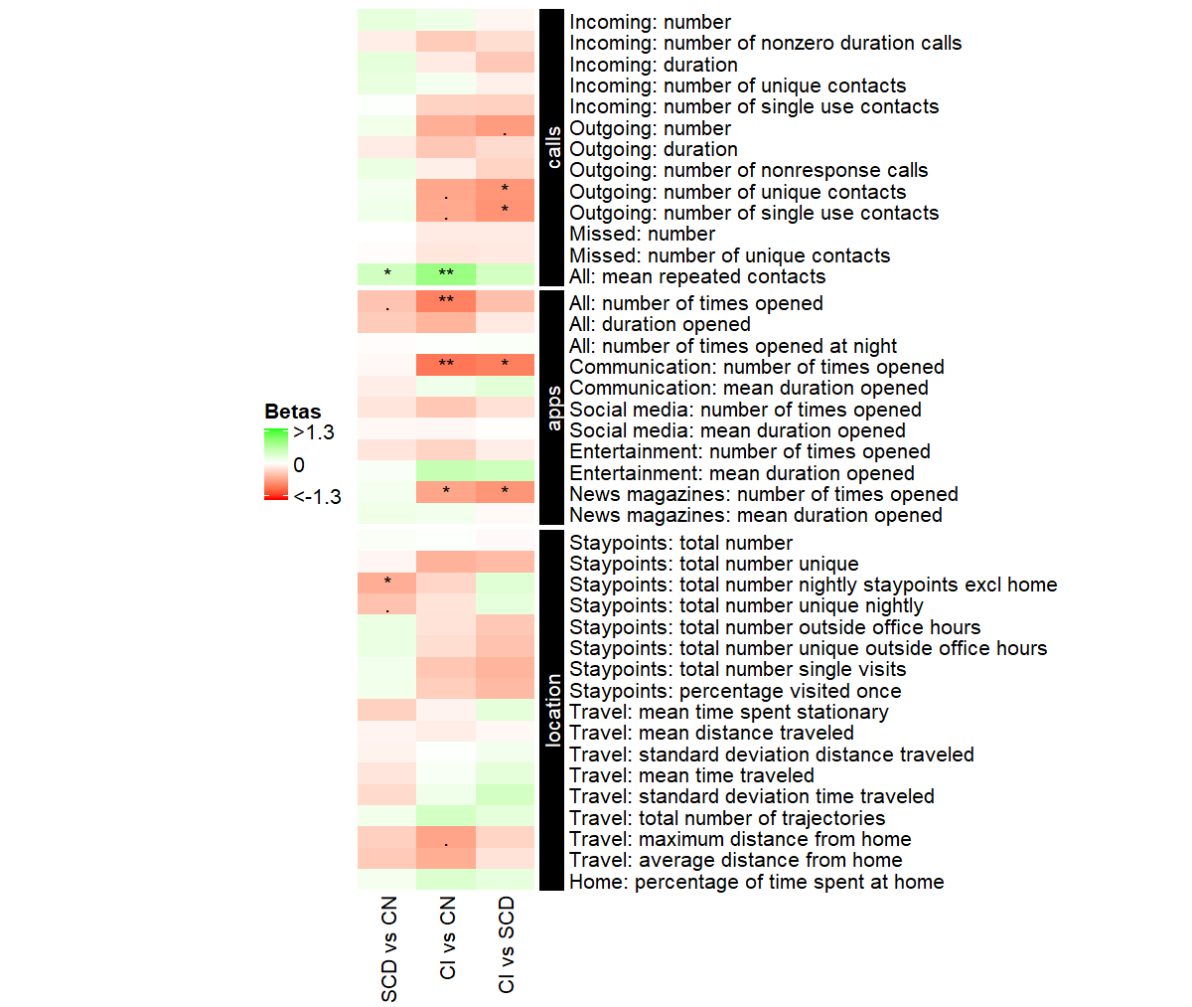
Differences in Behapp outcomes between the 3 diagnostic groups (cognitively impaired [CI], subjective cognitive decline [SCD], and cognitively normal [CN]) participants. Green squares indicate that the first mentioned group shows on average higher values on that Behapp outcomes than the second mentioned group. Red squares indicate that the first mentioned group shows on average lower values on that Behapp outcome than the second mentioned group. All analyses are corrected for age, sex, and education (ie, Behapp outcome ~ diagnostic group + age + sex + education). ** indicates *P*<.01; * indicates *P*<.05; . indicates *P*<.10, after correction for multiple comparisons. SCD: subjective cognitive decline; CN: cognitively normal; CI: cognitively impaired.

Compared with the CN and SCD participants, CI individuals had fewer unique outgoing contacts and contacted these same contacts more often. CI and SCD individuals both had higher scores in mean repeated contacts relative to CN (Figure 1, Table 3).

CI individuals made less use of apps compared with the CN participants. Compared with the CN and SCD groups, the CI group made less use of communication and news magazines apps (Figure 1, Table 3).

For the location variables, after correction for multiple comparisons, no differences were found between CI individuals and CN and SCD groups. Compared with CN individuals, SCD individuals visited fewer places at night excluding home (Figure 1, Table 3).

**Table 3:** Differences between diagnostic groups for each Behapp outcome.

Variable		SCD^a^ vs CN^b^, *β* (SE)	*P* value	CI^c^ vs CN, *β* (SE)	*P* value	CI vs SCD, *β* (SE)	*P* value	
**Calls**								
	Incoming: number	0.23 (0.14)	.30	0.16 (0.21)	>.99	–0.06 (0.24)	>.99	
	Incoming: number of nonzero duration calls	–0.12 (0.18)	>.99	–0.35 (0.27)	.61	–0.23 (0.31)	>.99	
	Incoming: duration (s)	0.24 (0.14)	.29	–0.14 (0.22)	>.99	–0.38 (0.25)	.39	
	Incoming: number of unique contacts	0.20 (0.14)	.46	0.10 (0.21)	>.99	–0.10 (0.24)	>.99	
	Incoming: number of single use contacts	0.02 (0.16)	>.99	–0.30 (0.24)	.68	–0.31 (0.28)	.80	
	Outgoing: number	0.13 (0.17)	>.99	–0.54 (0.25)	.10	–0.67 (0.29)	.06	
	Outgoing: duration (s)	–0.13 (0.18)	>.99	–0.37 (0.27)	.51	–0.24 (0.31)	>.99	
	Outgoing: number of nonresponse calls	0.18 (0.16)	.72	–0.11 (0.24)	>.99	–0.29 (0.27)	.83	
	Outgoing: number of unique contacts	0.10 (0.17)	>.99	–0.60 (0.25)	.06	–0.70 (0.29)	.049	
	Outgoing: number of single use contacts	0.14 (0.16)	>.99	–0.58 (0.24)	.05	–0.72 (0.28)	.03	
	Missed: number	0 (0.16)	>.99	–0.14 (0.24)	>.99	–0.14 (0.27)	>.99	
	Missed: number of unique contacts	–0.02 (0.16)	>.99	–0.17 (0.24)	>.99	–0.15 (0.27)	>.99	
	All: mean repeated contacts	0.41 (0.16)	.04	0.80 (0.25)	.004	0.39 (0.28)	.49	
**App use**								
	All: number of times opened	–0.40 (0.17)	.06	–0.83 (0.25)	.004	–0.43 (0.29)	.41	
	All: duration opened (s)	–0.35 (0.18)	.16	–0.50 (0.27)	.19	–0.15 (0.31)	>.99	
	All: number of times opened at night	–0.02 (0.16)	>.99	0.02 (0.23)	>.99	0.04 (0.27)	>.99	
	Communication: number of times opened	–0.04 (0.18)	>.99	–0.89 (0.27)	.004	–0.84 (0.31)	.02	
	Communication: mean duration opened (s)	–0.12 (0.17)	>.99	0.14 (0.27)	>.99	0.26 (0.30)	>.99	
	Social media: number of times opened	–0.17 (0.15)	.75	–0.38 (0.23)	.28	–0.21 (0.26)	>.99	
	Social media: mean duration opened (s)	–0.05 (0.19)	>.99	–0.06 (0.37)	>.99	–0.01 (0.40)	>.99	
	Entertainment: number of times opened	–0.18 (0.15)	.73	–0.29 (0.23)	.60	–0.11 (0.26)	>.99	
	Entertainment: mean duration opened (s)	0.06 (0.34)	>.99	0.49 (0.63)	>.99	0.43 (0.68)	>.99	
	News magazines: number of times opened	0.10 (0.16)	>.99	–0.60 (0.23)	.03	–0.70 (0.26)	.03	
	News magazines: mean duration opened (min)	0.15 (0.18)	>.99	0.11 (0.33)	>.99	–0.03 (0.36)	>.99	
**Location**								
	Total number of stay points	0.05 (0.19)	>.99	0.02 (0.25)	>.99	–0.03 (0.30)	>.99	
	Total number of unique stay points	–0.06 (0.18)	>.99	–0.52 (0.25)	.11	–0.46 (0.29)	.35	
	Total number of nightly stay points excluding home	–0.55 (0.20)	.02	–0.28 (0.27)	.89	0.27 (0.31)	>.99	
	Total number of unique nightly stay points	–0.41 (0.18)	.08	–0.18 (0.25)	>.99	0.23 (0.29)	>.99	
	Total number of outside office hours stay points	0.18 (0.18)	.89	–0.19 (0.24)	>.99	–0.38 (0.28)	.54	
	Total number of unique outside office hours stay points	0.19 (0.18)	.86	–0.22 (0.24)	>.99	–0.41 (0.28)	.43	
	Total number of single visits	0.11 (0.19)	>.99	–0.39 (0.25)	.38	–0.50 (0.30)	.28	
	Percentage of stay points visited once	0.13 (0.19)	>.99	–0.34 (0.25)	.56	–0.47 (0.30)	.36	
	Mean time spent stationary (min)	–0.31 (0.18)	.27	–0.08 (0.25)	>.99	0.23 (0.29)	>.99	
	Mean distance traveled (km)	–0.07 (0.17)	>.99	–0.12 (0.23)	>.99	–0.05 (0.28)	>.99	
	Standard deviation distance traveled (km)	–0.09 (0.18)	>.99	0.03 (0.24)	>.99	0.11 (0.29)	>.99	
	Mean time traveled (min)	–0.18 (0.17)	.93	0.07 (0.23)	>.99	0.24 (0.28)	>.99	
	Standard deviation time traveled (min)	–0.25 (0.18)	.49	0.14 (0.24)	>.99	0.39 (0.29)	.52	
	Total number of trajectories	0.13 (0.18)	>.99	0.38 (0.24)	.36	0.25 (0.29)	>.99	
	Maximum distance from home (km)	–0.32 (0.20)	.32	–0.61 (0.27)	.08	–0.29 (0.32)	>.99	
	Average distance from home (km)	–0.36 (0.19)	.20	–0.55 (0.27)	.12	–0.20 (0.31)	>.99	
	Percentage of time spent at home	0.10 (0.19)	>.99	0.32 (0.25)	.62	0.23 (0.30)	>.99	

^a^SCD: subjective cognitive decline.

^b^CN: cognitively normal.

^c^CI: cognitively impaired.

### Neuropsychiatric Symptoms

**Figure 2:**
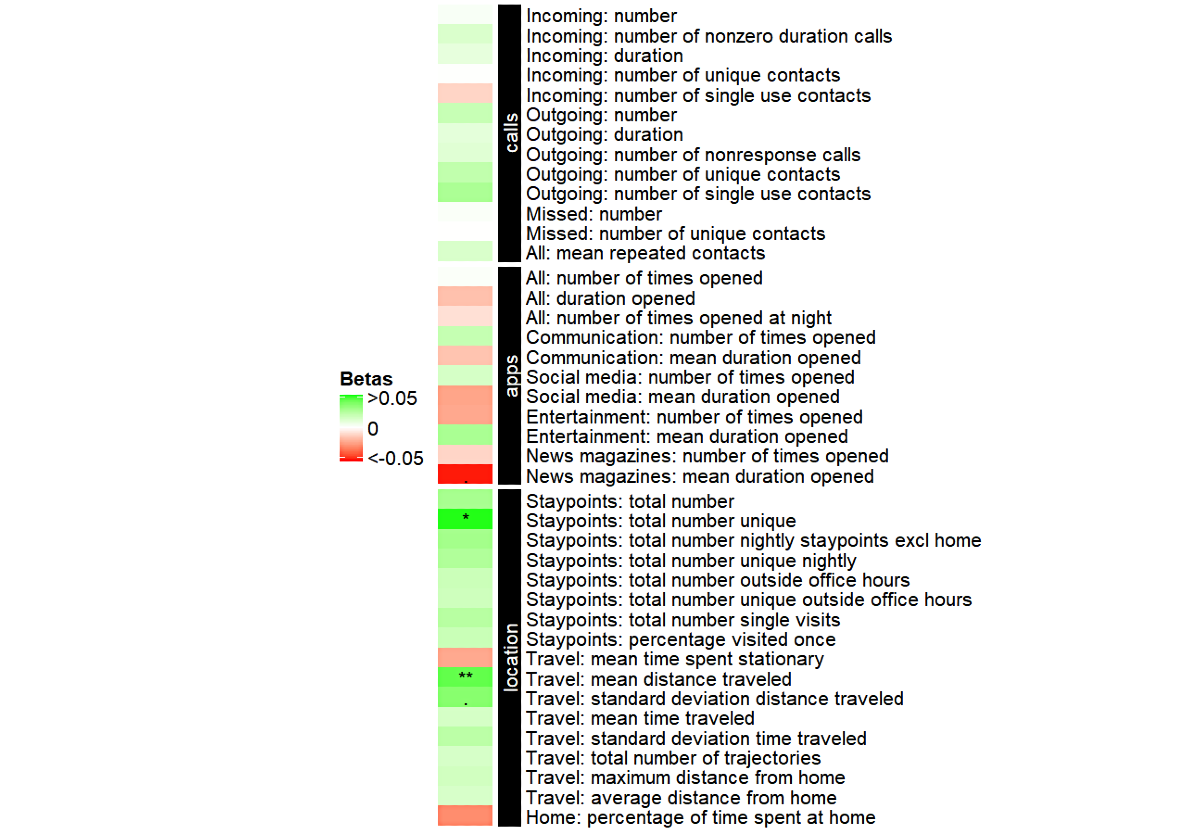
Association between Behapp outcomes and the neuropsychiatric inventory (NPI) total score. Green squares indicate that the Behapp outcome is positively related to the NPI, while red squares indicate that the Behapp outcome is negatively related to the NPI. All analyses are corrected for age, sex, and education (ie, Behapp outcome ~ NPI total score + age + sex + education). * indicates *P*<.05; . indicates *P*<.10, after correction for multiple comparisons.

Total NPI scores were available for 19 SCD participants and 22 CI participants. Scores did not differ between the groups (Table 1). In the combined sample, higher NPI total scores were associated with a higher mean distance traveled (Figure 2). Irritability, apathy, appetite, and depression were the most present neuropsychiatric symptoms in both the CI and SCD groups. When stratifying for these subscores, higher irritability scores were associated with longer use of news magazine apps and longer distance traveled (Multimedia Appendix 1, Table S2). We observed no other associations between Behapp outcomes and NPI subscores. Similar results were found when also correcting for diagnostic group.

## Discussion

### Principal Findings

The most important finding of this study to assess social behavior in CN and CI participants is that CI participants differ from CN and SCD individuals according to the signal generated by the passive monitoring app Behapp. Differences were especially found in variables showing repetitive and unique behavior.

In the CN control group, we found that older individuals called less frequently and made less use of apps. A possible explanation for this age effect is that older participants are overall less inclined to use their smartphone and make more use of traditional ways to communicate—for example, calling with their landline, reading a printed newspaper, or simply forgetting to take their phone when going out. Since this behavior cannot be registered with the Behapp app, our findings do not necessarily mean that older adults experience diminished social behavior. No clear pattern of associations with education was found. No sex effects were found, which was unexpected as women usually have larger social networks [14].

The most important Behapp outcomes to distinguish CI participants from CN and SCD participants were related to repetitive or unique social behavior: CI patients called more often with the same contacts. Although the CI group is significantly older, it is unlikely that the found effects can be explained by older age alone, since the total amount of calls, traveling, and visited places for each group is similar, and the analyses were corrected for age. This reduced exploratory behavior for CI patients is in line with previous studies that showed that individuals with CI had smaller social networks [27]. Furthermore, CI participants made less use of communication and news magazine apps, which suggests they are less socially engaged. However, since CI participants made less use of apps in general, these results should be interpreted with caution. Additionally, a trend was seen that CI patients travel less far from home compared to cognitively healthy participants. This is in accordance with previous findings with GPS trackers in multiple studies showing that the mobility range of Alzheimer disease patients is diminished [28,29]. SCD participants showed similar behavior patterns as the CN group, except for the number of nightly stay points. SCD is a heterogeneous condition [20], in which some may develop Alzheimer disease later on, but the presence of amyloid biomarkers was small in our sample and we therefore cannot compare preclinical Alzheimer disease to controls.

To our knowledge, no previous research is available about the association between social behavior as measured with a smartphone app and neuropsychiatric symptoms in an Alzheimer disease population. Since neuropsychiatric symptoms are frequently prevalent in Alzheimer disease patients [30] and multiple neuropsychiatric symptoms, such as depression, are related to social withdrawal [3], we expected to find associations between NPI scores and Behapp outcomes. However, we found that neuropsychiatric symptoms were associated with further distance traveled only in the combined SCD/CI group. A possible explanation for these findings is that overall scores were low, and consequently, the range of NPI scores was small. We observed some associations on subscores but these are difficult to interpret given the large number of tests.

### Comparison With Prior Work

One can argue whether Behapp is a proxy for social behavior, since the app does not capture offline communication. Especially in this older generation, interaction with other people is often face to face or calling with a landline. However, prior work shows a proof of principle that Behapp can capture changes in human behavior caused by an external factor, which in our case is the disease [12]. Other work shows an association of the Behapp outcomes with 2 questionnaires assessing social functioning and loneliness (in preparation). It is therefore assumed that the Behapp outcomes are a proxy for social behavior, albeit not the full range of social behavior, and are helpful to capture changes in social behavior.

### Strengths and Limitations

Despite our unique data set, large control group, and sufficient follow-up time, this study has some limitations. First, the Behapp app was not available on smartphones with an iOS operating system, which could lead to a selection bias. One participant received an Android phone for the duration of the study, but removal of this participant did not influence the results. Second, the Behapp app measures only one aspect of social functioning: on one hand, other forms of social contact are possible that cannot be measured with a smartphone such as meeting someone in person, and on the other, altered social behavior in Alzheimer disease patients does not automatically lead to subjective feelings of loneliness in these patients. The Behapp app only assesses communication via calls, while an increasing amount of communication is via social media apps. Because of privacy regulations, it is impossible to track the number of text messages sent with social media apps. We could therefore have missed important communication information. Further research should include questionnaires to identify methods of communication used and to assess loneliness. Third, mobility patterns of an individual are often influenced by their partner, especially when they are CI. The Behapp app only measured mobility patterns of the participant and did not take into account mobility patterns of possible partners or caregivers, which could explain why we did not find stronger associations. Fourth, another limitation is that the CI group consisted of individuals with both MCI and Alzheimer-type dementia. Since patients living with dementia experience by definition more difficulties with instrumental activities of daily living [31], effects could have been larger when stratifying analyses for these clinical groups. Besides this, the CI group was small, and therefore important associations could have been missed. Finally, the majority of participants in the CN and SCD groups did not receive an extensive neuropsychological assessment; their normal cognition is not objectified.

### Future Directions

Further research should focus on confirming our results with larger groups, with extensive neuropsychological assessment to confirm cognition status, and in longitudinal cohorts. We recommend using objective and passive smartphone apps in intervention studies aiming to diminish social withdrawal, using outcome variables measuring unique and repetitive behavior in particular.

### Conclusion

This research shows that the Behapp smartphone app is able to objectively and passively find differences between CI and CN participants. These findings provide support for the use of passive monitoring tools for characterizing altered social behavior in Alzheimer disease, although more research needs to be done.

## Appendix

Multimedia Appendix 1 Supplementary material.

## Abbreviations

CI: cognitively impaired
CN: cognitively normal
EFPIA: European Federation of Pharmaceutical Industries and Associations
IMI JU: Innovative Medicines Initiative 2 Joint Undertaking
MCI: mild cognitive impairment
NPI: Neuropsychiatric Inventory
PRISM: Psychiatric Ratings Using Intermediate Stratified Markers
SCD: subjective cognitive decline
