# Supplementary material for: Assessment of Social Behavior Using a Passive Monitoring App in Cognitively Normal and Cognitively Impaired Older Adults: Observational Study
Source: JMIR Aging. 2022 May 20;5(2):e33856. doi: 10.2196/33856 (PMC9166640; doi:10.2196/33856)
Supplement: Multimedia Appendix 1 [file aging_v5i2e33856_app1.docx]

## Multimedia Appendix 1. Supplementary material.

*
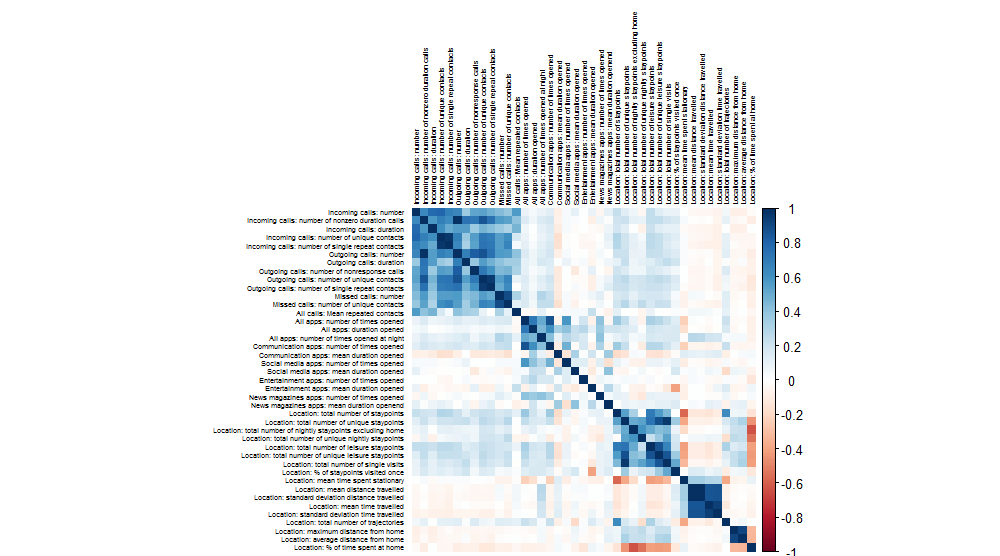
*

*Figure S1: Correlation matrix of the normalized BEHAPP outcome variables. Clear correlation patterns can be seen in the calls category, app usage category and location category.*

Table S1: Participant information for each cohort

| Hersenonderzoek.nl:   - Participants provided information on their demographics and health using the online Hersenonderzoek.nl platform. - Participants had to own an Android smartphone and use it on a daily basis. - CN participants self-indicated absence of neurological or psychiatric diseases without memory complaints - SCD participants self-indicated absence of neurological or psychiatric diseases with memory complaints. - CI participants self-indicated presence of AD |
| --- |
| ADC:   - Participants visited the Alzheimer Center Amsterdam because of cognitive complaints and underwent an extensive test battery of neuropsychological tests, physical examination, electroencephalography (EEG) and magnetic resonance imaging (MRI) scans [23]. - Participants had to own an Android phone and use it on a daily basis. - Participants had a Mini-Mental State Examination (MMSE) score higher than 20. - SCD participants visited the memory clinic with memory complaints but did not show objective cognitive deficits during neuropsychological testing. - CI participants were clinical diagnosed with either mild cognitive impairment or AD dementia. |
| PRISM:   - Participants were recruited in the Netherlands (Universitair Medisch Centrum Utrecht in Utrecht, Amsterdam Universitair Medisch Centrum in Amsterdam and Leids Universitair Medisch Centrum in Leiden) (n=19) and Spain (Hospital Gregorio Marañon and Hospital Universitario La Princesa, both in Madrid) (n=16). - One participant received an Android phone for the duration of the study since he owned an iPhone. - CN participants had to have an average MMSE score according to their age and years of education, as compared to normative data. - CI participants had to have a MMSE score between 20 and 26. - More detailed in- and exclusion criteria for the PRISM project can be found in Bilderbeck, et al. [24]. |

Table S2: The associations between the NPI total score, sub-scales and each BEHAPP variable.

| Variable | Total | Depression | | Apathy | | | Irritability | | Appetite |
| --- | --- | --- | --- | --- | --- | --- | --- | --- | --- |
| Calls | | | | | | | | | |
| **Incoming calls: number** | 0.002 (0.004),*P=*1 | | 0.013 (0.022),*P=*1 | | -0.004 (0.018),*P=*1 | -0.006 (0.019),*P=*1 | | 0.013 (0.016),*P=*1 | |
| **Incoming calls: number of nonzero duration calls** | 0.013 (0.025),*P=*1 | | 0.081 (0.146),*P=*1 | | 0.035 (0.123),*P=*1 | -0.007 (0.127),*P=*1 | | 0.039 (0.107),*P=*1 | |
| **Incoming calls: duration** | 0.009 (0.018),*P=*1 | | 0.049 (0.106),*P=*1 | | 0.007 (0.09),*P=*1 | -0.008 (0.092),*P=*1 | | 0.05 (0.077),*P=*1 | |
| **Incoming calls: number of unique contacts** | 0 (0.003),*P=*1 | | 0.012 (0.018),*P=*1 | | -0.011 (0.015),*P=*1 | -0.016 (0.015),*P=*.88 | | 0.003 (0.013),*P=*1 | |
| **Incoming calls: number of single use contacts** | -0.011 (0.03),*P=*1 | | 0.004 (0.172),*P=*1 | | -0.15 (0.143),*P=*.91 | -0.189 (0.146),*P=*.61 | | -0.032 (0.126),*P=*1 | |
| **Outgoing calls: number** | 0.019 (0.035),*P=*1 | | 0.115 (0.203),*P=*1 | | 0.041 (0.172),*P=*1 | -0.008 (0.178),*P=*1 | | 0.054 (0.149),*P=*1 | |
| **Outgoing calls: duration** | 0.009 (0.025),*P=*1 | | 0.066 (0.145),*P=*1 | | 0.017 (0.123),*P=*1 | -0.008 (0.127),*P=*1 | | 0.021 (0.107),*P=*1 | |
| **Outgoing calls: number of nonresponse calls** | 0.011 (0.021),*P=*1 | | 0.049 (0.119),*P=*1 | | -0.019 (0.1),*P=*1 | 0.024 (0.103),*P=*1 | | -0.058 (0.087),*P=*1 | |
| **Outgoing calls: number of unique contacts** | 0.02 (0.035),*P=*1 | | 0.112 (0.202),*P=*1 | | 0.056 (0.171),*P=*1 | 0.006 (0.177),*P=*1 | | 0.069 (0.149),*P=*1 | |
| **Outgoing calls: number of single use contacts** | 0.026 (0.03),*P=*1 | | 0.154 (0.172),*P=*1 | | 0.08 (0.147),*P=*1 | 0.074 (0.151),*P=*1 | | 0.113 (0.126),*P=*1 | |
| **Missed calls: number** | 0.002 (0.014),*P=*1 | | 0.034 (0.079),*P=*1 | | -0.052 (0.066),*P=*1 | -0.003 (0.069),*P=*1 | | 0.03 (0.058),*P=*1 | |
| **Missed calls: number of unique contacts** | 0 (0.014),*P=*1 | | 0.031 (0.08),*P=*1 | | -0.064 (0.066),*P=*1 | -0.019 (0.069),*P=*1 | | 0.029 (0.058),*P=*1 | |
| **All calls: mean repeated contacts** | 0.013 (0.026),*P=*1 | | 0.009 (0.149),*P=*1 | | -0.005 (0.125),*P=*1 | 0.059 (0.129),*P=*1 | | 0.047 (0.109),*P=*1 | |
| App usage | | | | | | | | | |
| **All apps: number of times opened** | 0.001 (0.021),*P=*1 | | 0.187 (0.115),*P=*.34 | | 0.126 (0.098),*P=*.63 | -0.085 (0.101),*P=*1 | | 0.042 (0.089),*P=*1 | |
| **All apps: duration opened** | -0.016 (0.023),*P=*1 | | 0.267 (0.127),*P=*.13 | | 0.045 (0.113),*P=*1 | -0.192 (0.111),*P=*.28 | | -0.031 (0.101),*P=*1 | |
| **All apps: number of times opened at night** | -0.008 (0.016),*P=*1 | | 0.09 (0.091),*P=*.98 | | -0.04 (0.077),*P=*1 | -0.086 (0.077),*P=*.83 | | -0.077 (0.068),*P=*.78 | |
| **Communication apps: number of times opened** | 0.019 (0.041),*P=*1 | | 0.142 (0.236),*P=*1 | | 0.084 (0.199),*P=*1 | 0.001 (0.203),*P=*1 | | 0.102 (0.177),*P=*1 | |
| **Communication apps: mean duration opened** | -0.015 (0.021),*P=*1 | | 0.128 (0.117),*P=*.85 | | -0.055 (0.1),*P=*1 | -0.13 (0.1),*P=*.60 | | -0.053 (0.089),*P=*1 | |
| **Social media apps: number of times opened** | 0.014 (0.021),*P=*1 | | 0.174 (0.119),*P=*.46 | | 0.071 (0.103),*P=*1 | -0.003 (0.105),*P=*1 | | -0.006 (0.092),*P=*1 | |
| **Social media apps: mean duration opened** | -0.023 (0.018),*P=*.63 | | -0.089 (0.108),*P=*1 | | 0.063 (0.103),*P=*1 | -0.102 (0.095),*P=*.89 | | -0.054 (0.087),*P=*1 | |
| **Entertainment apps: number of times opened** | -0.022 (0.019),*P=*.72 | | -0.076 (0.11),*P=*1 | | 0.075 (0.092),*P=*1 | -0.136 (0.092),*P=*.44 | | 0.035 (0.083),*P=*1 | |
| **News magazines apps: number of times opened** | -0.011 (0.02),*P=*1 | | 0.092 (0.113),*P=*1 | | -0.101 (0.095),*P=*.89 | -0.09 (0.097),*P=*1 | | -0.025 (0.086),*P=*1 | |
| **News magazines apps: mean duration opened** | -0.049 (0.025),*P=*.20 | | -0.177 (0.136),*P=*.63 | | 0.151 (0.153),*P=*1 | -0.438 (0.137),*P=*.01 | | -0.03 (0.119),*P=*1 | |
| Location | | | | | | | | | |
| **Total number of stay points** | 0.027 (0.025),*P=*.83 | | -0.01 (0.142),*P=*1 | | -0.014 (0.122),*P=*1 | 0.119 (0.125),*P=*1 | | 0.222 (0.109),*P=*.16 | |
| **Total number of unique stay points** | 0.049 (0.02),*P=*.07 | | 0.202 (0.119),*P=*.30 | | -0.019 (0.108),*P=*1 | 0.159 (0.109),*P=*.47 | | 0.211 (0.096),*P=*.11 | |
| **Total number of nightly stay points excluding home** | 0.029 (0.025),*P=*.77 | | 0.197 (0.137),*P=*.49 | | 0.134 (0.12),*P=*.81 | 0.172 (0.123),*P=*.53 | | 0.117 (0.116),*P=*.96 | |
| **Total number of unique nightly stay points** | 0.025 (0.02),*P=*.66 | | 0.185 (0.11),*P=*.31 | | 0.046 (0.099),*P=*1 | 0.09 (0.102),*P=*1 | | 0.098 (0.094),*P=*.91 | |
| **Total number of outside office hours stay points** | 0.018 (0.021),*P=*1 | | 1 | | 0.108 (0.103),*P=*.91 | 0.082 (0.108),*P=*1 | | 0.067 (0.1),*P=*1 | |
| **Total number of unique outside office hours stay points** | 0.017 (0.021),*P=*1 | | 0.074 (0.121),*P=*1 | | 0.096 (0.103),*P=*1 | 0.076 (0.108),*P=*1 | | 0.062 (0.1),*P=*1 | |
| **Total number of single visits** | 0.023 (0.03),*P=*1 | | 0.097 (0.168),*P=*1 | | 0.136 (0.143),*P=*1 | 0.099 (0.151),*P=*1 | | 0.087 (0.139),*P=*1 | |
| **% of stay points visited once** | 0.018 (0.03),*P=*1 | | 0.077 (0.17),*P=*1 | | 0.146 (0.144), *P=*.96 | 0.085 (0.152),*P=*1 | | 0.065 (0.141),*P=*1 | |
| **Mean time spent stationary** | -0.022 (0.024),*P=*1 | | 0.023 (0.139),*P=*1 | | 0.06 (0.119),*P=*1 | -0.096 (0.123),*P=*1 | | -0.211 (0.108), *P=*.18 | |
| **Mean distance travelled** | 0.042 (0.015), *P=*.03 | | 0.045 (0.097),*P=*1 | | 0.1 (0.081),*P=*.68 | 0.302 (0.065), *P=*.0002 | | 0.103 (0.078),*P=*.58 | |
| **Standard deviation distance travelled** | 0.035 (0.018),*P=*.19 | | -0.021 (0.108),*P=*1 | | 0.124 (0.09),*P=*.54 | 0.295 (0.079), *P=*.003 | | 0.082 (0.088),*P=*1 | |
| **Mean time travelled** | 0.014 (0.013),*P=*.82 | | -0.078 (0.072),*P=*.87 | | -0.049 (0.063),*P=*1 | 0.082 (0.064),*P=*.63 | | 0.134 (0.055),*P=*.07 | |
| **Standard deviation time travelled** | 0.022 (0.017),*P=*.60 | | 0.024 (0.097),*P=*1 | | 0.023 (0.083),*P=*1 | 0.137 (0.083),*P=*.33 | | 0.131 (0.076),*P=*.29 | |
| **Total number of trajectories** | 0.014 (0.019),*P=*1 | | 0.07 (0.109),*P=*1 | | 0.05 (0.094),*P=*1 | 0.111 (0.096),*P=*.78 | | 0.083 (0.09),*P=*1 | |
| **Maximum distance from home** | 0.016 (0.023),*P=*1 | | 0 (0.128), *P=*1 | | 0.216 (0.103),*P=*.14 | 0.125 (0.112),*P=*.82 | | 0.018 (0.106),*P=*1 | |
| **Average distance from home** | 0.014 (0.023),*P=*1 | | 0.008 (0.13),*P=*1 | | 0.202 (0.106),*P=*.21 | 0.113 (0.115),*P=*1 | | -0.015 (0.108),*P=*1 | |
| **% of time spent at home** | -0.029 (0.024),*P=*.71 | | -0.221 (0.132),*P=*.32 | | 0.046 (0.119),*P=*1 | -0.119 (0.122),*P=*1 | | -0.114 (0.113),*P=*.96 | |

*The second column shows the betas (SE) and significance levels from the regression model: BEHAPP outcome ~ NPI total score score + Age + Sex + Education. The third to sixth columns show the betas (SE) and significance levels from 4 separate regression models for the four most present NPI sub-scales (BEHAPP outcome ~ NPI subscale score + Age + Sex + Education). Significant results are highlighted in green, after correction for multiple testing. Abbreviations: SE, standard error; NPI, Neuropsychiatric Inventory.*
